# HIV-1 Envelope Conformation, Allostery, and Dynamics

**DOI:** 10.3390/v13050852

**Published:** 2021-05-07

**Authors:** Ashley Lauren Bennett, Rory Henderson

**Affiliations:** 1 Duke Human Vaccine Institute, Durham, NC 27710, USA; ashley.bennett@duke.edu; 2 Department of Medicine, Duke University, Durham, NC 27710, USA

**Keywords:** HIV-1, envelope, allostery, molecular dynamics, structure

## Abstract

The HIV-1 envelope glycoprotein (Env) mediates host cell fusion and is the primary target for HIV-1 vaccine design. The Env undergoes a series of functionally important conformational rearrangements upon engagement of its host cell receptor, CD4. As the sole target for broadly neutralizing antibodies, our understanding of these transitions plays a critical role in vaccine immunogen design. Here, we review available experimental data interrogating the HIV-1 Env conformation and detail computational efforts aimed at delineating the series of conformational changes connecting these rearrangements. These studies have provided a structural mapping of prefusion closed, open, and transition intermediate structures, the allosteric elements controlling rearrangements, and state-to-state transition dynamics. The combination of these investigations and innovations in molecular modeling set the stage for advanced studies examining rearrangements at greater spatial and temporal resolution.

## 1. Introduction

**Figure 1:**
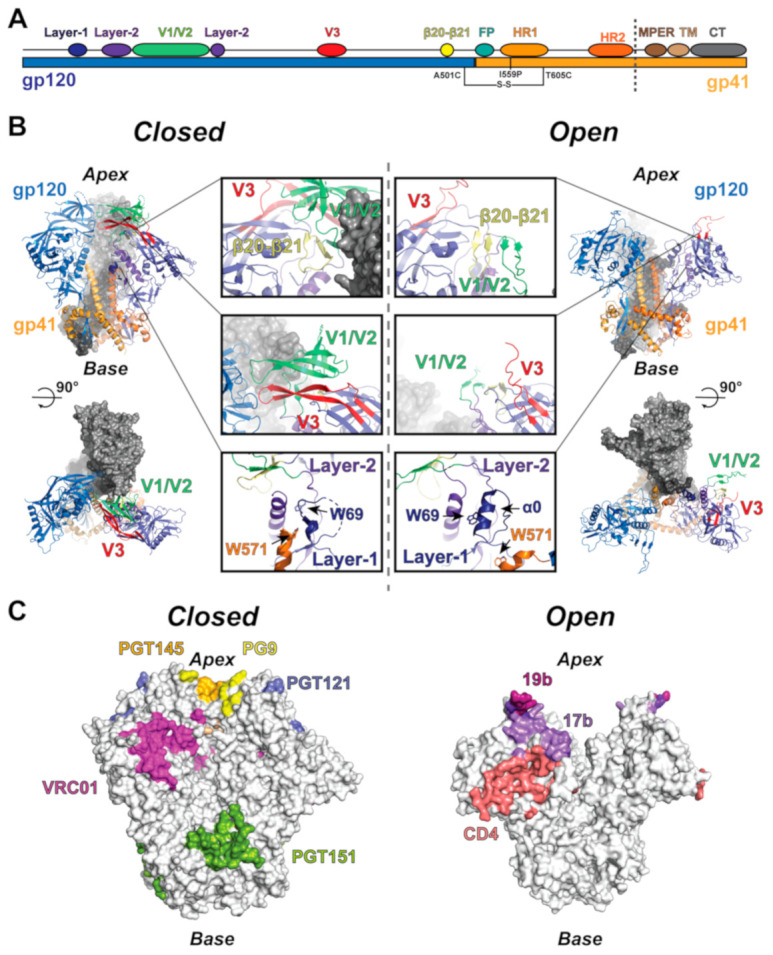
Structure and allosteric elements of the HIV-1 Env trimer. (**A**) Linear depiction of the HIV-1 Env structural elements highlighting the position of the SOSIP mutations and the soluble ectodomain region (to dashed line). (**B**) (upper left) Side view of the closed state trimer. The protomer to the left is colored according to the gp120 and gp41 domains, while the protomer to the right is colored according to allosteric elements, including the β20–β21 loop (yellow), V1/V2 (lime), V3 (red), layer-1 (dark blue), and layer-2 (purple). (lower left) Top view of the closed state trimer depicting the apex gp120 contacts. (middle left) Closed state allosteric elements. (middle right) Open state allosteric elements. (top right) Side view of the open state trimer colored as the closed state trimer. (lower right) Top view of the open state trimer depicting the broken apex contacts. (**C**) Closed and open state surfaces highlighting the epitopes of HIV-1 Env-targeting antibodies.

The heavily glycosylated HIV-1 envelope (Env) glycoprotein mediates host cell fusion and represents an important target for vaccine immunogen design. The Env is a trimer of identical subunits, each composed of an N-terminal 120 kDa domain, termed gp120, and a C-terminal 41 kDa domain, termed gp41, separated by a proteolytic cleavage site [1]. The transmembrane gp41 domain contains the conserved fusion elements [2,3] and is protected from immune surveillance by close association with gp120. The gp120 domains contain the receptor and co-receptor binding sites [4,5], which, upon CD4 receptor engagement, undergo a series of conformational transitions (Figure 1) [4,5]. These conformational rearrangements expose the co-receptor binding site and fusion machinery, ultimately leading to the fusion of the viral membrane with the host cell [4,5,6]. This intricate process has been the subject of intense study, as the control of Env immunogen conformation has a direct impact on epitope exposure, and therefore, immunization outcomes [7,8,9,10,11,12].

The marked mutability of the HIV-1 virus presents a significant challenge to immune responses as epitope contacts shift during infection, rendering most neutralizing antibody responses specific to a particular Env sequence [13]. This is exacerbated by the fact that mutation often alters the conformational preferences of the Env. Despite these obstacles, antibodies capable of binding to and neutralizing diverse HIV-1 isolates, termed broadly neutralizing antibodies (bnAbs), have been identified in infected individuals [14,15]. These bnAbs preferentially recognize a closed state of HIV-1 Env [16,17,18,19,20] in which the gp120 subunits form close interactions with the internal, conserved gp41 fusion elements and with one another at the trimer apex. This highly protected state forces potentially neutralizing antibodies to penetrate and traverse the Env glycan shield to reach their target epitope [21,22]. The HIV-1 Env is capable of transitioning between open, closed, and intermediate states in the absence of its receptor [7,19,20,23], a process that is often referred to as Env “breathing” [23,24,25,26]. This phenomenon is susceptible to mutation, resulting in different Env isolates having differing preferences and propensities for the sampling of these different states [17,18,19,20,27]. 

Studies examining Env prefusion conformations and structural rearrangements associated with transitions observed upon CD4 and small molecule binding have provided a detailed view of Env dynamics. These investigations have taken advantage of X-ray crystallography [8,11,28,29,30,31,32,33,34,35,36,37,38,39,40,41,42,43,44,45,46,47,48,49,50] and advances in cryo-electron microscopy (cryo-EM) [7,9,24,25,48,51,52,53,54,55,56,57,58,59,60,61,62,63,64,65,66,67], single-molecule techniques [18,19,20,27], and high-precision, residue-specific, pairwise distance measures [17], in combination with mutagenesis and biophysical characterization. Where techniques such as cryo-EM can provide high-resolution snapshots of particularly stable states and single-molecule methods can provide detailed transition kinetics and equilibrium state probabilities, bridging the resolution and timescale gaps of these methods for interrogation of Env metastability increasingly relies upon a hybrid approach, combining computational studies with experimental output. Here, we review experimental and computational methods aimed at interrogating Env conformational dynamics at the earliest stages of triggering and provide an overview of theoretical approaches capable of shedding light on the processes underlying transitions between states of the molecule.

## 2. Env Structure, Allostery, and Dynamics

Structures of the HIV-1 Env trimer are primarily derived from soluble ectodomain constructs composed of the full gp120 domain and the segment of gp41 N-terminal to the membrane external proximal region (MPER), transmembrane helices, and C-terminal domain (CT) [7,68]. These ectodomain constructs are typically stabilized by the mutagenic introduction of a disulfide bond (SOS) between gp120 and gp41 near the trimer base in addition to a helix-breaking isoleucine-to-proline (IP) mutation in the gp41 N-terminal heptad repeat 1 (HR1), referred to together as the SOSIP construct (Figure 1A,B) [12,69]. Many Env ectodomain crystal and cryo-EM structures, both alone and in complex with various antibodies, receptors, and small molecules, have been determined [7,8,11,24,25,28,29,30,31,32,33,34,35,36,37,38,39,42,44,46,47,48,49,50,51,52,53,54,55,59,60,61,62,63,64,66,67]. These include bnAbs such as quaternary specific antibodies PGT145 and PGT151 [9,24,56,57,58,59], variable loops 1/2 (V1/V2) targeting PG9 [60], CD4-binding site-targeting VRC01 [28,50,70,71,72,73], V3-glycan targeting PGT121 [11], and bridging-sheet targeting 17b [25,31,51,55,74,75], as well as the CD4 receptor [25,51,55,59] (Figure 1C), the closed state locking BMS compounds [34,49], and open state-inducing BMN-III-170 and M48U1 compounds [74]. Additionally, several detergent-solubilized full-length and CT truncated structures have been determined, revealing overall topological similarity between the SOSIP and full-length trimers [9,56,57,58,76,77]. The closed state ectodomain structure is characterized by a trimer apex at which the gp120 domains contact. The contact points occur between a region containing two loops whose sequences vary at significantly greater rates compared to other regions of the Env [5], termed variable loops one and two (V1 and V2) as well as a third variable loop (V3) that packs closely against V1/V2 [78,79] (Figure 1A,B). The gp120 domain itself is split into a receptor-binding outer domain, containing the V1/V2 and V3 loops, as well as two other variable loops (V4 and V5), and a gp41-contacting inner domain [5]. Together, the gp41 domains form an internal three-helix bundle protected by the adjacent gp120s with the C-terminal portion of each gp41 wrapping around the gp120 termini, forming the base of the trimer [80,81] (Figure 1A,B). In structures of antibody and CD4 stabilized open and intermediate states, the gp120 displays a rotation about a hinge near its gp41-contacting termini that, in the open state, is accompanied by rotation of the gp41 base [25,51,55,70]. These rearrangements are accompanied by multiple within-domain tertiary structure rearrangements that are associated with receptor interaction.

Multiple structural states of the HIV-1 Env have been identified, including a prefusion closed state, several antibodies stabilized intermediate states, and a receptor-bound open state [25,51,55,69,82]. Triggering of the prefusion closed state is initiated at a CD4 receptor-binding loop, termed β20–β21. Engagement by CD4 results in the movement of this loop, causing a cascade of rearrangements in gp120 that ultimately lead to the open state in which gp120 contacts with internal gp41 elements are broken [25]. This is accompanied by the disengagement of V1/V2 and V3 from the primary outer domain structure. Additional rearrangements occur in a region of gp120 termed the inner domain layers [5,25,51,55]. The innermost layer, layer-1, contacts the first heptad repeat of gp41 (HR1) and is surrounded by a second layer, layer-2, which is made up of a helical segment, α1, and a loop segment [25]. In the closed state, layer-1 residue W69 is buried between the layer-2 segments. In the open state, W69 is displaced from this position, with layer-1 acquiring helical content to form helix α0, which packs against the loop segment of layer-2 [25,83]. The β20–β21 loop in the open state acquires an anti-parallel β-sheet configuration, pairing with anti-parallel β2 and β3, forming what is referred to as the bridging sheet [25,51,55]. These movements together are associated with the displacement of the V1/V2 region and the V3 loop to apparently disordered states. This eliminates gp41 HR1 W571 contact with the layer-1 to layer-2 α1 helix interface, permitting extension of the C-terminus of the HR1 helix and resulting in the rotation of gp120 away from HR1 about an axis near the second gp41 heptad repeat, HR2 [25,51,55,70]. Mutations limiting movements in β20-β21 [8,30,35,37,59,84], V3 [10,12,35,40,70], and the inner domain layers [8,10,70] have all proven effective in preventing transitions from the prefusion state. Together, these rearrangements and their ability to guide closed state locking mutations define the allosteric elements that work together to sense receptor engagement and allow the exposure of gp41 fusion elements.

The structural dynamics and conformational heterogeneity of multiple virion-associated, full-length HIV-1 Env isolates have been explored extensively using single-molecule Förster resonance energy transfer (smFRET) [18,19,20,27]. This technique allows for monitoring of the distance between two amino acid-linked dye molecules placed at different positions on a macromolecule. In the case of the Env, these positions included pairings in V1 and V4, V1 and V5, and V1 and gp41. Information gleaned from these investigations has provided millisecond–second timescale state transition kinetics, estimates of transition probabilities [18,19,20,27], state occupancies [18,19,20,27], and the CD4-induced changes in conformational free energy and activation barriers [27] for HIV-1 Env isolates NL4-3 [18,19,20], JR-FL [18,19,20,27], and BG505 [18,27]. Experiments performed with the V1/V4 dye pairing in native, virion-associated [18,19,20,27] Env constructs demonstrated that the Env favors a low FRET ground state, indicating the dyes, and therefore the dye-conjugated amino acids, are typically distant from one another [18,19,20,27]. The unliganded Env was observed to spontaneously sample intermediate and high FRET states [18,19,20,27], with the high FRET state acting as a transition intermediate for exchange between low and intermediate FRET states. The relative propensity to occupy these states differed among the isolates and was sensitive to small molecule, receptor, and antibody binding as well as the addition of stabilizing mutations [18,19,20,27,30,70]. Data gathered for the BG505 SOSIP Env construct displayed reduced low FRET state occupancy with a concomitant increase in high FRET state occupancy relative to the virion-associated Env [27]. The binding of CD4 and 17b increases the occupancy of the intermediate FRET state, indicating this state represents the CD4-bound, open Env (Figure 1C) [18,19,20,27]. The low FRET state is preferred by HIV-1 bnAbs, such as apex-targeting PGT145 and CD4-binding site-targeting VRC01 (Figure 1C), and higher occupancy of the low FRET state is correlated with virus neutralization resistance, suggesting it corresponds to a closed state structure [49]. However, differences in the relative occupancies of states between full-length, membrane-associated and soluble ectodomain Envs leaves some question as to the structural relationship between the smFRET measured states and available high-resolution structures [27].

Additional solution state amino acid position details for the SOSIP Env construct were provided by double electron–electron resonance (DEER) spectroscopy. This method provides pairwise distance measurements between specifically labeled amino acid positions. The predominant interprotomer distances observed between V1/V2, V3, the bridging sheet, the inner domain, and gp41 allosteric elements in the unliganded HIV-1 Env BG505 and B41 SOSIPs [17] were comparable to those observed in closed state SOSIP [11,33,34,36,38] HIV-1 Env structures. In contrast to the conformational heterogeneity observed in smFRET experiments for the unliganded state of HIV-1 Env [18,19,20,27], significant structural heterogeneity in the V1/V2, V3, or bridging sheet elements was not observed in these DEER experiments. Both Env SOSIPs were sensitive to CD4 receptor and antibody binding with subtle differences in their position preferences. While observations were overall consistent with the available structural data, several novel distances were observed, particularly in gp41, indicating that Env accesses additional, uncharacterized conformational states [17]. Together, the smFRET and DEER experiments reveal a dynamic Env structure for which unobserved states remain to be interrogated at the atomic scale.

## 3. Molecular Motions in the HIV-1 Env: A Theoretical Perspective

While considerable experimental data have identified specific sites of allosteric control of the HIV-1 Env trimer conformation, these approaches are generally limited to an equilibrium, ensemble-based or low-resolution description of the relevant transitions. A complete transition mechanism describing the order in which the Env-triggering elements move, with identification of the short-lived transition states that determine the state-to-state transition kinetics, remains elusive. Experimental limitations in spatial and temporal resolution necessitate alternative approaches. One such method is molecular dynamics (MD) simulation. These simulations use a combination of Newtonian mechanics and a simplified representation of atomic interactions to describe molecular motions [85]. As a complement to structural data collected from crystallographic and cryo-EM techniques, MD can provide a detailed description of time-dependent molecular motions, allowing for the identification of transition states at the atomic scale that are difficult to observe experimentally. Thus, MD can act as a bridge between low-resolution data from mutagenesis or single-molecule methods and high-resolution structural data. Simulations of the HIV-1 Env, its subunits, and its domains, are beginning to shed light on these details. A key consideration for MD analyses is the simulation timescale that is computationally accessible. A typical *in silico* atomistic system of a fully glycosylated HIV-1 Env trimer contains between 500 K and 1 M atoms, which, on currently available GPU hardware, requires roughly one to two days to complete a 50-nanosecond simulation [86]. With smFRET indicating that the relevant, major transitions occur on timescales of milliseconds to seconds, simulating a single closed-to-open state transition could therefore take years to observe. Simulations have therefore focused on either short-timescale phenomena or have taken advantage of approaches that increase the probability of observing such transitions. 

The dense glycan shield of the Env has been the subject of several MD-based investigations, as the glycans tend to sample configurational space at a comparatively quick rate. The glycan shield helps to obscure bnAb epitopes in the closed state HIV-1 Env, thereby protecting the Env from immune recognition [28]. Due to intrinsic glycan mobility, X-ray crystal and cryo-EM structures are limited in their ability to resolve individual glycans, necessitating this alternative approach toward their characterization. Several studies have examined N-linked glycans modeled on the surface of both the open gp120 monomer [87] and closed state trimer [28,88,89], including a non-MD, integrated cryo-EM modeling study [90]. In the case of simulation, marked structural heterogeneity in the glycan shield structure [28,90,91] showed that glycan dynamics contribute to overall glycoprotein structural stability [87,90]. Transient glycan–glycan interactions have also been observed, resulting in the formation of stable glycan clusters in addition to close, transient interactions with the CD4 receptor when present [28,92]. These HIV-1 Env glycan shield simulations have also identified the presence of glycan “holes” in which portions of the Env surface are exposed due to shield conformational heterogeneity [91,92]. Finally, simulation has revealed that the shield can effectively compensate for the loss of a glycan via modification of the glycan interaction network, [88,90], explaining how the loss of a glycan distant from the binding site can increase bnAb neutralization resistance [93]. These simulations provide a detailed atomistic view of the glycan structural variability, adding a layer of complexity to our understanding of the shield dynamics and its influence on bnAb activity.

Where the glycan shield’s conformational dynamics occur on timescales amenable to currently available computer code and hardware, investigations of the Env conformation must be tailored to specific questions. One such study investigated a fully glycosylated, closed state BG505 SOSIP trimer during a two microsecond MD simulation, revealing the interprotomer motions of the gp120 domains [89]. Principal component analysis (PCA) identified an asymmetric scissoring motion of the individual protomers and accompanying symmetrical movements of gp120 protomers away from the trimer axis, occurring on sub-microsecond timescales [89], that are consistent with the cryo-EM structural results [56,59]. These sub-microsecond scissoring motions were observed to obscure the CD4bs bnAb epitopes, suggesting they play a role in CD4 binding and in virus neutralization sensitivity to bnAbs [89]. As the system size plays a major role in determining the total simulated time per compute time, truncated systems are often used to investigate transitions of interest. Several studies have made use of a single Env gp120 domain to investigate intradomain conformational dynamics [94,95,96]. These studies revealed gp120 is more conformationally variable in the CD4-bound state than in the unliganded state [94,95,96]. Similar to glycan dynamics, the protein loop segments often sample configurational space at a rate amenable to interrogation by standard MD. In examining the V3 loop, both MD [97] and a related conformational sampling technique, Monte Carlo simulation [78,79], suggested its release may occur without the dislocation of V1/V2 away from the trimer apex. Further, in another simulation-based investigation, spontaneous sampling of the closed state V3 was observed when simulations were initiated from the open state [97], suggesting this transition occurs on relatively short timescales. Together, these results suggest V3 exposure is not a rate-limiting factor in the initiation of the triggering event. Though binding of CD4 to the β20–β21 loop is believed to initiate the conformational cascade, another MD study indicated structural changes in V3 could lead to the rearrangement of the β20–β21 loop [98,99], suggesting more than one transition pathway may exist. Finally, a network-based analysis of correlated motions in gp120 monomers from multiple clades demonstrated amino acid communication pathways that differ subtly between clades [94]. This is consistent with the observed differences in the conformational distributions observed for different HIV-1 Env isolates in smFRET [18,19,27] and DEER [17] experiments. The results from the molecular dynamics simulation complement observations from experimental studies, identifying considerable glycan networking and predicting a complex allosteric communication network at temporal and structural resolutions amenable to rational vaccine and drug design.

## 4. Discussion

The HIV-1 envelope’s structure and dynamics have been the focus of intense investigation owing to its critical role in the virus lifecycle and its importance as an immunogenic target for vaccines. A wealth of structural data from X-ray crystallography and cryo-EM, smFRET, and DEER, in combination with biophysical interrogation of various mutants and Env isolates, has provided detailed mapping of the relevant structural features defining and regulating the initial conformational transitions the Env undergoes upon receptor CD4 engagement. These movements set the stage for fusion-specific rearrangements involving Env clustering [100,101,102], gp120 shedding [100,103,104], and six-helix bundle, coiled coil formation [3,105]. Together, high-resolution details of the allosteric network, along with an understanding of the dynamic nature of the transitions the Env undergoes, have proven effective in guiding vaccine immunogen design. This information has been used to stabilize the Env closed state to prevent off-target, non-neutralizing antibody responses via vaccination [8,10,12,30,35,37,40,43,46,59,70,84,106] and has provided insight into the mechanism by which small molecules inhibit transitions [34,49]. Nevertheless, much remains to be understood regarding the transitions between these early states at the atomic scale and with a finer temporal resolution. Recent advances in GPU hardware and molecular dynamics simulation techniques provide a means by which to interrogate these transitions in this manner, with many such investigations providing a backdrop from which to begin. Where simulation has thus far revealed extensive glycan dynamics and correlated motion in various allosteric elements within particular conformational states, a complete transition mechanism for movement between the closed, intermediate, and open states remains to be determined. As these transitions occur on the millisecond or greater timescale, more advanced simulation techniques will be required. Two such methods proved successful in monitoring conformational transitions in the SARS-CoV-2 Spike fusion protein involving its receptor-binding domain: adaptive sampling and weighted-ensemble path sampling [107,108]. These methods use ensembles of simulations to aid in the selection of initiation points for additional simulations to improve sampling of low-probability regions of the conformational space. In this way, these simulations increase the likelihood of observing rare transitions such as the closed-to-open transition of the HIV-1 Env. Alternatively, as the transition rate for the closed-to-open transition of the Env gp120 is likely much faster than that of the full trimer, a detailed examination of the internal mechanics of the Env allosteric elements in this construct may prove beneficial and informative for preparing systems and techniques for transitions in the full Env ectodomain trimer. Indeed, the nature of the intricate internal rearrangements necessary to initiate HIV-1 Env transitions may, in fact, require a hybrid experimental/computational approach, leveraging observations from and predictions of experimental outcomes from mutagenesis to guide the selection of competing models for the full transition. Such investigations will provide detailed information for understanding the nature of the transition states that govern movement between the major states already observed, and would have an important impact on our understanding of virus evolution as well as in drug development and immunogen design.

## Data Availability

Not applicable.
